# Potentiality of α-fetoprotein (AFP) and soluble intercellular adhesion molecule-1 (sICAM-1) in prognosis prediction and immunotherapy response for patients with hepatocellular carcinoma

**DOI:** 10.1080/21655979.2021.1990195

**Published:** 2021-12-10

**Authors:** Weiwei Cao, Yu Chen, Wei Han, Juzheng Yuan, Weimin Xie, Kun Liu, Yan Qiu, Xudan Wang, Xiao Li

**Affiliations:** a Department of Hepatobiliary Surgery, Xijing Hospital, Fourth Military Medical University, Xi’an, Shaanxi, People’s Republic of China; b Medical School , Ankang Vocational and Technical College, Ankang, Shaanxi, People’s Republic of China; c Department of General Surgery, Air Force 986th Hospital, Xi’an, Shaanxi, People’s Republic of China; d Department of Oncology Surgery, Shaanxi Provincial People’s Hospital, Xi’an, Shaanxi, People’s Republic of China; e Department of Hepatobiliary Surgery, Shenmu Hospital, Shenmu, Shaanxi, People’s Republic of China

**Keywords:** α-fetoprotein, soluble intercellular adhesion molecule-1, prognosis prediction, immunotherapy response

## Abstract

The α-fetoprotein (AFP) and soluble intercellular adhesion molecule-1 (sICAM-1) have certain diagnostic value, but their potential value in prognosis prediction, especially immunotherapy response prediction, remains unclear in liver cancer. Through the tumor-free survival (TFS) and overall survival (OS) rates analyses of serum AFP and sICAM-1 levels in 87 patients with primary hepatocellular carcinoma (HCC), the patients whose AFP and sICAM-1 levels were normal (AFP < 20 μg/L or sICAM-1 < 1000 μg/L) before surgery or recovered to normal after surgery exhibited a lower tumor recurrence rate and better OS than patients with elevated serum levels of the two markers. Combined analysis showed that patients with synchronously elevated levels of AFP and sICAM-1 showed the lowest TFS and OS. In addition, the RNA-seq data and clinical information of The Cancer Genome Atlas Liver Hepatocellular Carcinoma were collected to analyze the predictive values of AFP and ICAM-1 in the diagnosis, prognosis and immunotherapy of HCC. The results indicated that the combined application of the two indicators had higher accuracy in both the diagnosis and prognostic prediction of HCC by receiver operating characteristic curves. AFP and ICAM-1 were significantly correlated with multiple immune cells in HCC samples but not in normal samples. The patients with low expression of the two indicators were most likely to benefit from the immune checkpoint blockade therapy. In conclusion, AFP and ICAM-1 play vital roles in the diagnosis, prognostic prediction, and immunotherapy of HCC, suggesting that they are considered as prognostic predictors in clinical practice.

## Introduction

Primary hepatocellular carcinoma (HCC) has become one of the most significant malignant digestive neoplasms, due to the increasing number of patients with viral hepatitis, fatty hepatitis and alcoholic liver disease, threatening human health [1]. With the technological development of comprehensive treatment for liver cancer, postoperative survival conditions of patients with primary HCC have gradually improved. However, the newly diagnosed patients of liver cancer worldwide are still as many as 750,000 cases each year at present, and the patients of deaths are approximately 700,000 cases [2]. Such a high mortality rate directly leads to liver cancer becoming the second leading cause of cancer death in adults. The reason is closely related to the lack of effective diagnosis and treatment measurements [3]. Sensitive tumor markers contribute to correctly evaluating the malignant behaviors of tumors and accurately predicting the survival situations of patients with an early diagnosis, in addition to delaying tumor progression [4]. They play a vital role in the diagnosis and treatment of tumors. Therefore, searching for effective tumor markers is very urgent for the diagnosis and treatment of liver cancer.

Both the α-fetoprotein (AFP) and soluble intercellular adhesion molecule-1 (sICAM-1) are commonly used serum markers, especially in the diagnosis of primary HCC [5–8]. The sICAM-1 is a circulating form of ICAM-1, and serum sICAM-1 level can reflect the local expression of ICAM-1. Recent studies have found that AFP and ICAM-1 also play an important role in tumor prognosis. For example, a review of gastric cancer research suggested the effect of AFP on the prognosis of patients with gastric cancer [9]. Zhu *et al* suggested that the high level of ICAM-1 affected prognosis of patients with HCC [10]. However, whether AFP and ICAM-1 have a synergistic effect on the prognosis of HCC is still unknown.

AFP and ICAM-1 have multiple biological functions, for example, recent studies have reported that AFP not only plays a biological role as a tumor marker but also has the ability to regulate cell proliferation, differentiation, and tumorigenesis [11]. Similarly, ICAM-1 can drive inflammatory responses and it-mediated inflammation of immune cells plays a vital role in tumorigenesis [12]. In view of the multiple functions of AFP and ICAM-1, we speculate that they may be involved in the immunomodulation and immunotherapy of tumors. The study found the antitumour effect of AFP occurs mainly through redirecting human T cells to specifically recognize and kill HCC tumor cells without significant toxicity to normal primary hepatocytes *in vitro* [13]. Similarly, research on bladder cancer has suggested that high levels of sICAM-1 seem to increase the susceptibility of tumor cells to death by activating lymphocytes, and it can also improve the therapeutic efficacy of Bacillus Calmette-Guerin vaccine in the immunotherapy of bladder cancer [14]. These studies have shown the potential role of AFP and ICAM-1 in tumor immunomodulation, but they have not conducted multifaceted assessments to determine whether AFP and ICAM-1 can be potential targets for HCC immunotherapy.

In this study, on the one hand, we collected serum AFP and sICAM-1 levels of pre- and postoperative patients to assess their relationship with liver cancer recurrence and survival rate. On the other hand, we used bioinformatics methods to evaluate the potential value of AFP and ICAM-1 in HCC immunotherapy. We speculated that AFP and ICAM-1 can predict the prognosis of HCC patients and participate in immunotherapy of HCC. This study aimed to explore the clinical value of AFP and ICAM-1 to judge surgical prognosis and evaluate potential therapeutic effects for patients with liver cancer.

## Data & methods

### General information

The 87 patients with primary HCC who were diagnosed at Xijing Hospital (Xi’an, China) and treated with radical hepatectomy from May 2017 to December 2017. Among them, there were 61 male cases (71%) and 26 female cases (29%), with a male to female ratio of 2.34:1. The patients’ ages receiving surgery ranged from 29 to 78 (67.5 ± 8.9) years. Baseline liver diseases included 70 cases of chronic hepatitis B (80.4%), 51 cases of liver cirrhosis (58.6%), 8 cases of alcoholic hepatitis (9.2%), 4 cases of hepatitis C (4.6%) and 4 cases of other liver diseases (4.6%). According to the Child-Pugh classification, there were 43 cases (49.4%) of Child-Pugh class A, 25 cases of Child-Pugh class B (28.7%) and 19 cases of Child-Pugh class C (21.9%). Eighteen cases (20.7%) were stage I, 26 cases (29.9%) were stage II, 24 cases (27.6%) were stage III and 19 cases (21.8%) were stage IV, according to the tumor size/lymph nodes/distance metastasis (TNM) staging. For treatment, all cases received surgical resection. All cases presented with no residual tumor cells in the hepatic tissues surrounding the cancer focus by the confirmation of postoperative pathological examination. This study was approved by the Institutional Ethics Committee of First Affiliated Hospital of Fourth Military Medical University (Approval number: KY20172013-1), and written informed consent was obtained from each participant.

### Follow-up data

Follow-up was performed via outpatient recheck or phone call. The follow-up period was 18–66 months, with no patients lost to follow-up. All patients’ AFP and sICAM-1 levels were examined one month after surgery. Computed tomography or magnetic resonance imaging was applied once every 6 months to define the local or systemic condition of the patients. During the follow-up, a newly detected focus by imaging examination was predicated as tumor recurrence.

### Tumor markers detection

#### Sample collection

A total of 4 mL of blood from each patient was extracted in the morning 1 week preoperatively and one month postoperatively with the patient fasting. Samples were centrifuged at 3000 rpm for 10 minutes to obtain serum after standing undisturbed for 30 minutes.

#### AFP detection

Measuring instruments (AxSYM Automatic Chemiluminescence Analyzer) and the original matched reagents were supported by the Abbott Laboratories (Abbott, CHI, USA). The normal reference value is < 20 µg/L.

#### sICAM-1 detection

An ICAM-1 (Soluble) Human ELISA Kit was purchased from the Thermo Fisher Scientific Corporation (Thermo Fisher Scientific, MA, US). The procedure was performed according to the manufacturer’s protocol. The normal reference value is < 1000 µg/L.

### Cases grouping

First, patients were grouped according to the preoperative level of AFP or sICAM-1 and were classified into three groups: AFP < 20 µg/L, 20–200 µg/L and > 200 µg/L or sICAM-1 < 1000 µg/L, 1000–2000 µg/L and > 2000 µg/L. Then, the pre- and postoperative levels of the tumor markers were combined, and patients were classified into three groups: Group N: Both tumor markers were in the range of the normal reference value both pre- and postoperatively; Group D: the levels of the markers preoperatively were above the upper limit of the normal reference value but fell back to the normal range postoperatively; Group UI: the levels of the markers preoperatively were normal but rose above the upper limit of the normal reference value postoperatively, or the levels above the upper limit of the normal reference value preoperatively but rose, remained unchanged, or remained above the upper limit of the normal reference value, despite going down postoperatively. Third, all the patients were divided into four groups according to the combination of preoperative levels of two tumor markers: Group A: AFP < 20 µg/L and sICAM-1 < 1000 µg/L; Group B: AFP > 20 µg/L and sICAM-1 < 1000 µg/L; Group C: AFP < 20 µg/L and sICAM-1 > 1000 µg/L; Group E (to make it different from Group D above): AFP > 20 µg/L and sICAM-1 > 1000 µg/L.

### The predictive value of AFP and ICAM-1 in the diagnosis and prognosis of HCC

The transcriptome data and clinical data of 371 HCC samples and 50 normal samples were downloaded from TCGA database (https://portal.gdc.cancer.gov/). The diagnostic effect of AFP or ICAM-1 alone was analyzed by the pROC package (1.17.0.1 version) and visualized by the ggplot2 package in R (3.6.3 version) [15]. The *glm* function was used to construct a logistics model to assess the diagnostic effect of the combination of AFP and ICAM-1 [16].

The prognostic value of AFP or ICAM-1 alone was analyzed by the survivalROC package in HCC samples and normal samples [15]. A least absolute shrinkage and selection operator (LASSO) Cox regression model was constructed by the glmnet package in R to evaluate the prognostic value of the combination of AFP and ICAM-1 [17] .

The larger the area under the curve (AUC) value in the receiver operating characteristic curve (ROC) is, the better the prediction effect. The 0.5 < AUC < 1 indicates that there is predictive value.

### Immune cell infiltration analysis

The percentage abundance of tumor infiltrating immune cells was analyzed by the xCell algorithm using the immunedeconv package and visualized by the ggplot2 and pheatmap packages in R [18]. The significance of the three groups passed the Kruskal-Wallis test. The immune score, stromal score and microenvironment score among the groups were visualized by the ggplot2 package. The significance of the three groups passed one-way analysis of variance (ANOVA), and the intergroup comparison employed the Student-Newman-Keuls (SNK) – q test.

The correlation heatmaps of AFP and ICAM-1 with immune cells were plotted by the psych package in HCC samples and normal samples, respectively, and the correlation was calculated by Pearson’s correlation.

### Prediction of immunotherapy response

Based on expression profile data from TCGA, the expression of immune checkpoint-relevant genes, including SIGLEC15, IDO1, CD274, HAVCR2, PDCD1, CTLA4, LAG3 and PDCD1LG2 [19,20], was analyzed and visualized by the ggplot2 package in R. The significance of the three groups passed the Kruskal-Wallis test.

The Tumor Immune Dysfunction and Exclusion (TIDE) algorithm was used to predict the responsiveness of a single sample to immune checkpoint inhibitors (http://tide.dfci.harvard.edu/login/) [21]. Immune checkpoint blockade (ICB) therapy response prediction was executed by ImmuCellAI (http://bioinfo.life.hust.edu.cn/ImmuCellAI#!/). The TIDE score was high, ICB therapy had poor efficacy, and the survival period was short after receiving ICB treatment. The plots were drawn by ggplot2 (3.3.3 version) and ggpubr (0.4.0 version) in R. The significance of the two groups passed the Wilcox test.

### Statistical analysis

Statistics were analyzed using Statistic Package for Social Science (SPSS) 14.0 statistical software. T tests and Wilcoxon tests were used to compare two groups, and square variance analysis and one-way ANOVA were used to compare more than two groups. The SNK-q test was used for intergroup comparison. The χ2 test or Fisher’s exact probability was used to compare enumeration data. We compared between groups with the χ2 section method. Survival analysis was performed using the Kaplan-Meier method, and the log-rank test was used for intergroup comparisons. Pearson correlation analysis was employed to calculate the correlation coefficient of the two tumor markers and the immune cells. Two-tailed P < 0.05 was defined as statistically significant.

### Results

Although it had been proved that AFP and ICAM-1 had clinical value in the diagnosis of HCC, their potential value in prognosis prediction and immunotherapy response had not yet been fully explored. Based on the clinical analysis of 87 patients with primary HCC, we found that the patients, who had normal AFP and sICAM-1 levels (AFP < 20 μg/L or sICAM-1 < 1000 μg/L) before surgery or higher preoperative levels dropping to normal after surgery, had lower tumor recurrence rate and higher OS than patients with elevated serum level of the two markers. Further, the bioinformatics analysis showed that the combined application of the two indicators had higher accuracy in both diagnosis and prognostic prediction of HCC, and the patients with low-expression of the two indicators were most likely to benefit from ICB therapy.

### The relationship between preoperative levels of AFP and sICAM-1 and postoperative tumor recurrence and survival rate

**Figure 1.**
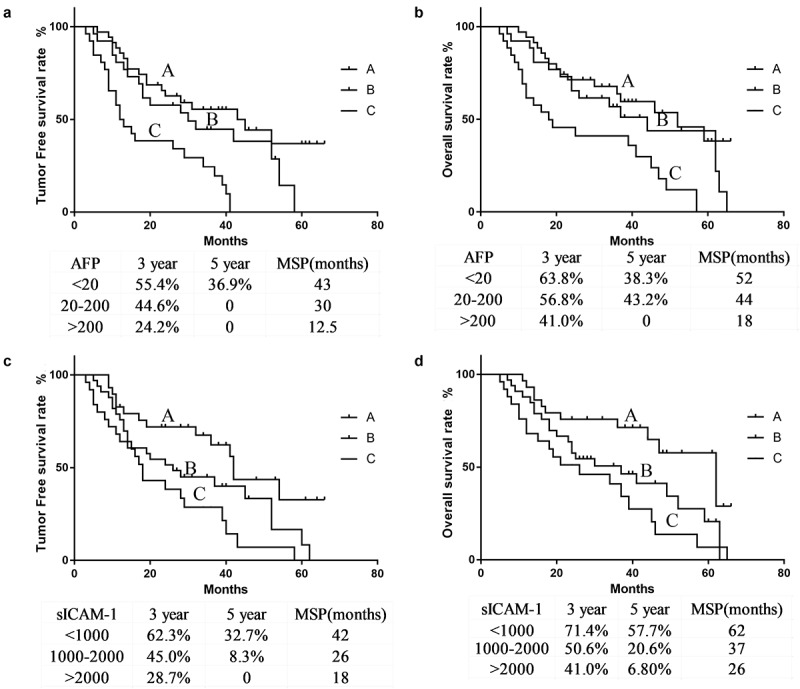
Postoperative tumor recurrence and overall survival rate for preoperative tumor markers. The tumor-free survival rate (a) and overall survival rate (b) of the patients with preoperative AFP < 20 µg/L (A), AFP 20–200 µg/L (B), and AFP > 200 µg/L (c). The tumor-free survival rate (**C**) and overall survival rate (d) of the patients with preoperative sICAM-1 < 1000 µg/L (A), sICAM-1 1000–2000 µg/L (B), and sICAM-1 > 2000 µg/L (C)

As shown in Table 1 and Figure 1, compared to patients with preoperative AFP < 20 μg/L, those with AFP > 200 μg/L had a higher tumor recurrence rate postoperatively (88.4% vs. 51.4%), lower 3-year or 5-year tumor-free survival rates (24.2%, 0% vs. 55.4%, 36.9%) and 3-year or 5-year overall survival rates of 41.0%, 0% vs. 63.8%, 38.3%, as well as shorter median tumor-free survival times (12.5 months vs. 43 months) and median overall survival times (18 months vs. 52 months).

**Table 1.** Relationship of patient tumor free rate in different tumor marker

Group	None recurrence	Recurrence	χ^2^	P
AFP(μg/L)			9.41	0.009
< 20	17	18		
20–200	8	18		
> 200	3	23		
sICAM-1(μg/L)			8.44	0.014
< 1000	15	14		
1000–2000	9	24		
> 2000	4	21

As shown in Table 2 and Figure 1, compared to patients with preoperative sICAM-1 > 2000 μg/L, those with sICAM-1 < 1000 μg/L had a lower tumor recurrence rate postoperatively (48.2% vs. 84.0%), higher 3-year or 5-year tumor-free survival rate (62.3%, 32.7% vs. 28.7%, 0%) and 3-year or 5-year overall survival rate 71.4%, 57.7% vs. 41.0%, 6.8%, as well as longer median tumor-free survival time and median overall survival time (42 m, 62 m vs. 18 m, 26 m) .

**Table 2.** Relationship of patient Overall survival rate in different tumor marker

Group	Survival	Death	χ^2^	P
AFP(μg/L)			7.96	0.019
< 20	19	16		
20–200	9	17		
> 200	5	21		
sICAM-1(μg/L)			8.98	0.011
< 1000	17	12		
1000–2000	11	22		
> 2000	5	20		

### The correlation between postoperative levels of AFP and sICAM-1 and clinicopathological indicators

Table 3 and Table 4 summarize the correlation between postoperative levels of AFP and sICAM-1 and clinicopathological indicators. In terms of postoperative levels of AFP, the 87 patients could be divided into Group N (25 cases), Group D (44 cases), and Group UI (18 cases). The preoperative levels of sICAM-1 could be divided into Group N (21 cases), Group D (46 cases), and Group UI (20 cases). Statistical analysis suggested that larger tumor lesions, more advanced TNM staging and a higher rate of tumor recurrence were mainly distributed in Group UI, whether in the AFP or sICAM-1 grouping. The recurrence rate of AFP-N group was 48%, AFP-D group 70.5%, and AFP-UI group 88.9%. The recurrence rate of sICAM-1-N group was 57.1%, sICAM-1-D group 76.1%, and sICAM-1-UI group 80%.

**Table 3.** Expression of AFP and the pathological indicators

Pathological indicators	N group 25	D Group 44	UI Group 18	P value
Gender				0.663
Male	17	32	12	
Female	8	12	6	
Age	67 ± 9	65 ± 8	64 ± 10	0.229
Child stage				0.397
Stage A	13	21	9	
Stage B	7	13	5	
Stage c	5	10	4	
HBV infection				0.306
Positive	18	32	13	
Negative	7	12	5	
Tumor number				0.333
Single	15	31	13	
Multiple	10	13	5	
Tumor size				0.016
< 2 cm	12	14	2	
2 cm – 5 cm	7	17	6	
> 5 cm	6	13	10	
TNM stage				0.026
Stage I	8	8	2	
Stage II	8	14	4	
Stage III	6	13	5	
Stage IV	3	9	7	
Tumor recurrence				0.025
None	13	13	2	
Recurrence	12	31	16

**Table 4.** Expression of sICAM-1 and the pathological indicators

Pathological indicators	N group	D Group	UI Group	P value
Gender				0.602
Male	14	32	15	
Female	7	14	5	
Age	63 ± 11	66 ± 10	67 ± 11	0.161
Child stage				0.034
Stage A	13	28	2	
Stage B	5	11	9	
Stage c	3	7	9	
HBV infection				0.895
Positive	16	33	14	
Negative	5	13	6	
Tumor number				0.603
Single	13	33	13	
Multiple	8	13	7	
Tumor size				<0.001
< 2 cm	13	11	4	
2 cm – 5 cm	6	17	7	
> 5 cm	2	18	9	
TNM stage				0.024
Stage I	11	5	2	
Stage II	6	13	7	
Stage III	3	16	5	
Stage IV	1	12	6	
Tumor recurrence				0.032
None	9	15	4	
Recurrence	12	31	16	

### The relationship between the change in AFP and sICAM-1 and tumor-free and overall survival rate

**Figure 2.**
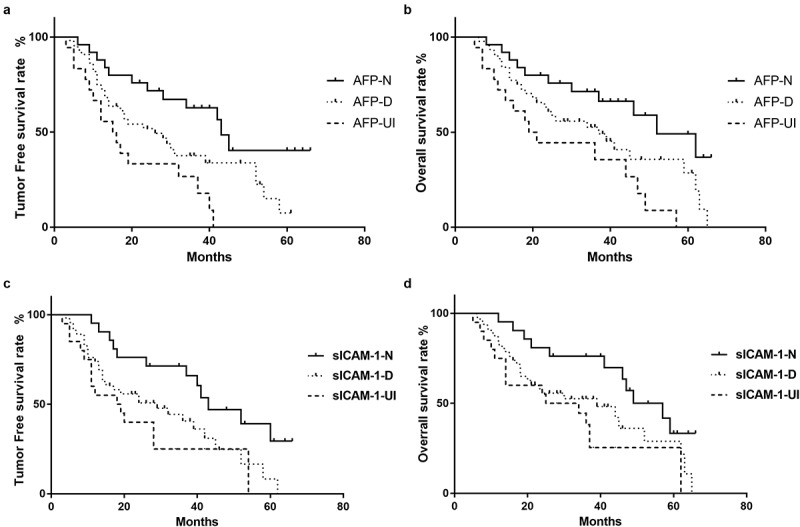
Relationship between the change in tumor markers and tumor-free survival and overall survival rates. The tumor-free survival rate (a) and overall survival rate (b) in the AFP-N group, AFP-D group, and AFP-UI group. The tumor-free survival rate (c) and overall survival rate (d) in the sICAM-1-N group, sICAM-1-D group, and sICAM-1-UI group

As indicated in Table 5 and Figure 2(a), the 3-year and 5-year tumor-free survival rates of the AFP-N group were 62.8% and 40.3%, respectively, which were significantly better than those of the AFP-UI group (26.7%, 0%, P < 0.001) and AFP-D group (37.9%, 7.5%, P = 0.033). As shown in Table 6 and Figure 2(b), the 3-year and 5-year overall survival rates of the AFP-N group (71.3%, 49.3%) were significantly higher than those of both the AFP-D group (52.6%, 28.61%) and AFP-UI group (35.6%, 0) (P = 0.049, P = 0.002). Meanwhile, the 3-year and 5-year tumor-free survival rates (71.4%, 29.4%) and 3-year and 5-year overall survival rates (76.2%, 33.3%) of the sICAM-1-N group remained significantly higher than those of the sICAM-1- UI group (25.0%, 0%, P = 0.005; 38.1%, 25.4%, P = 0.011) (Figure 2(c,d), Table 6).

**Table 5.** Tumor free survival rate according to the change of tumor marker AFP and sICAM-1

Group	3 year	5 year	P	Mean tumor free survival period (month)
AFP				
N Group	62.8%	40.3%		43
D Group	37.6%	7.5%	0.033	26
UI Group	26.7%	0	<0.001	15.5
sICAM-1				
N Group	71.4%	29.4%		43
D Group	40.7%	8.3%	0.018	29
UI Group	25%	0	0.005	18

**Table 6.** Overall survival rate according to the change of tumor marker AFP and sICAM-1

Group	3 year	5 year	P	Mean overall survival period (month)
AFP				
N Group	71.3%	49.3%		52
D Group	52.6%	28.6%	0.049	37
UI Group	35.6%	0	0.002	20
sICAM-1				
N Group	76.2%	33.3%		53
D Group	52.5%	28.9%	0.054	39
UI Group	38.1%	25.4%	0.011	29.5

### The patient survival rate according to the combination of preoperative levels of AFP and sICAM-1

**Figure 3.**
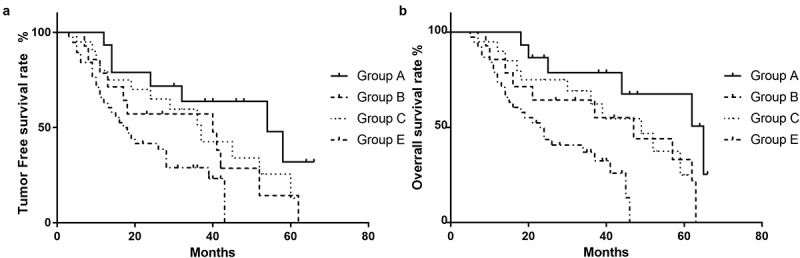
Tumor-free and overall survival according to the combination of markers. The tumor-free survival rate (a) and overall survival rate (b) in Group A, Group B, Group C, and Group E

According to the grouping strategy above, patients were divided into four groups: Group A contained 15 patients, Group B contained 14 patients, Group C contained 20 patients and Group E contained 38 patients, respectively. The mean tumor-free survival period of each group was 50, 40, 37, and 17 months, respectively (Table 7). Survival analysis demonstrated that the tumor-free survival rate of Group A was the highest, with 3-year and 5-year rates of 63.8% and 31.9%, respectively, which were significantly higher than those of Group E (with 3-year and 5-year rates of 28.9% and 0%, respectively) (P = 0.004). As shown in Figure 3(a), there were no statistically significant differences in tumor-free survival rates between Group A and Groups B and C, but there were trends to be different (P = 0.071; P = 0.205). Group E had the lowest tumor-free survival rate, which was significantly lower than Groups A and C (P < 0.001; P = 0.007), and there was a trend to be lower than Group B (P = 0.141). Similarly, as shown in Table 8 and Figure 3(b), the mean overall survival period of each group was 65 47, 49, and 23 months, respectively. Survival analysis demonstrated that Group A (the 3-year and 5-year overall survival rates were 78.8% and 67.5%) had a significantly higher rate than Group E (with rates of 37% and 0%) (P = 0.001), and Group E had the worst overall survival when compared with Groups A, B and C (P = 0.001; P = 0.042; P = 0.007). However, the differences between Groups A, B and C needed more investigation (P > 0.05) (Figure 3(b)).

**Table 7.** Tumor free survival rate with combination of preoperative AFP and sICAM-1

Group	Tumor free survival rate			Mean tumor free survival period (month)
	3 year	5 year	P	
Group A	63.8%,	31.9%,		50
Group B	57.1%	14.3%	0.071	40
Group C	51.1%	12.7%	0.205	37
Group E	28.9%	0	0.04	17

**Table 8.** Overall rate with combination of preoperative AFP and sICAM-1

Group	Total survival rate			Mean tumor free survival period (month)
	3 year	5 year	P	
Group A	78.8%	67.5%		65
Group B	64.3%	33.1%	0.060	47
Group C	62.3%	24.9%	0.118	49
Group E	37%	0%	0.001	23

### Identification of predictive values of AFP and ICAM-1 in the diagnosis and prognosis of patients with HCC

**Figure 4.**
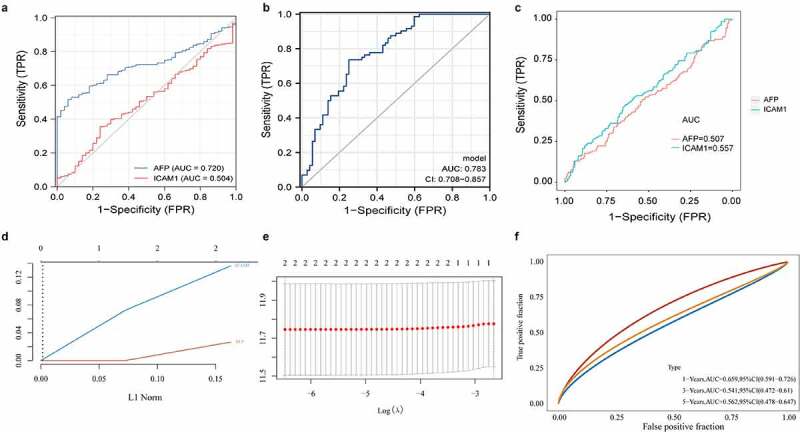
The predictive values of AFP and ICAM-1 in the diagnosis and prognosis of HCC. The ROC curves of AFP (a), ICAM-1 (**A**), and their combination (b) to predict the diagnostic value of the two markers in HCC. A logistics model was constructed by the *glm* function. The ROC curves of AFP and sICAM-1 (c) to predict the prognostic value of the two markers in HCC. A LASSO Cox regression model (d and e) was constructed by the glmnet package in R. The time-dependent ROC curve of the combination (f) was used to evaluate the prognostic value of AFP and sICAM-1

Based on 371 HCC samples and 50 normal samples with matched transcriptome data and clinical data from TCGA database, ROC curves were plotted using AFP and ICAM-1 expression data to identify their ability to recognize HCC samples and normal samples. We found that the recognition capability of AFP combined with ICAM-1 (AUC _combination_ = 0.783, the model was constructed by the *glm* function, Figure 4(b)) was higher than that of AFP alone (AUC _AFP_ = 0.720, AUC _ICAM1_ = 0.504, Figure 4(a)). Additionally, the survival data of all samples from TCGA database were employed to evaluate the ability of AFP and ICAM-1 to distinguish the prognosis of patients with HCC and normal subjects. The results indicated that both AFP (AUC _AFP_ = 0.507, Figure 4(c)) and ICAM-1 (AUC _ICAM1_ = 0.557, Figure 4(c)) had predictive value for the prognosis of patients with HCC. To further evaluate the predictive value of AFP combined with ICAM-1 with regard to patient prognosis, we first constructed a LASSO Cox regression model (Figure 4(d) and Figure 4(e)) and then plotted the time-dependent ROC curve of the model. The results showed that the predictive value of the model at 1 year (AUC _combination_ = 0.659) and 5 years (AUC _combination_ = 0.562) was higher than that of AFP or ICAM-1 alone with regard to patient prognosis (figure 4(f)). Taken together, these findings suggested the high diagnostic value and high prognostic value of the combined predictive factors.

### The relationship of AFP and ICAM-1 with the tumor microenvironment (TME)

**Figure 5.**
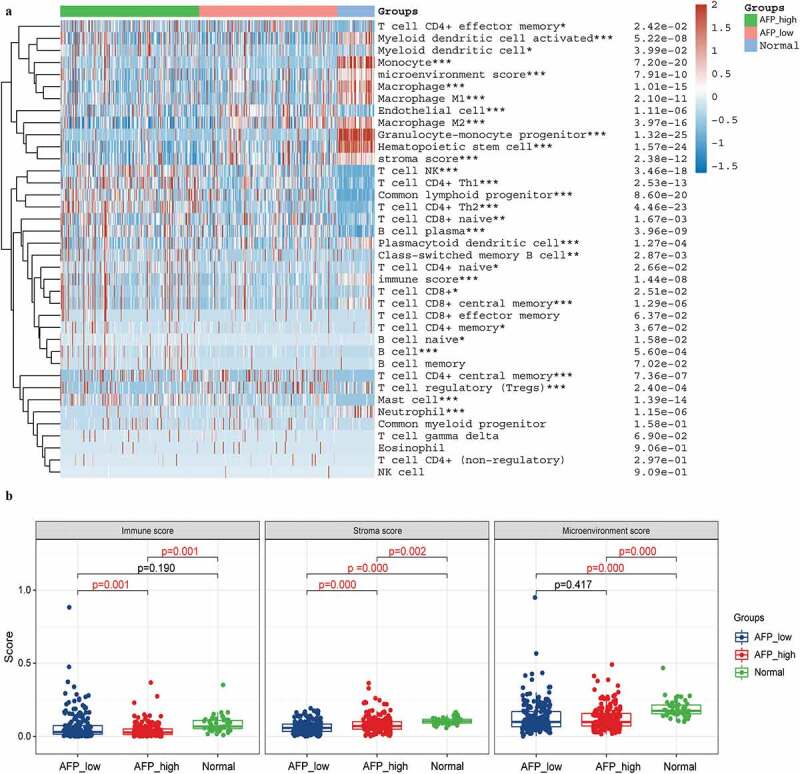
Immune cell infiltration analysis in AFP_high, AFP_low and normal samples. (a) The percentage abundance of tumor-infiltrating immune cells was analyzed by the xCell algorithm using the immunodeconv package and visualized by the ggplot2 and pheatmap packages in R. The significance of the three groups passed the Kruskal-Wallis test. (b) The immune score, stromal score and microenvironment score were visualized by the ggplot2 package. The significance of the three groups passed one-way ANOVA. P < 0.05 was defined as statistically significant

**Figure 6.**
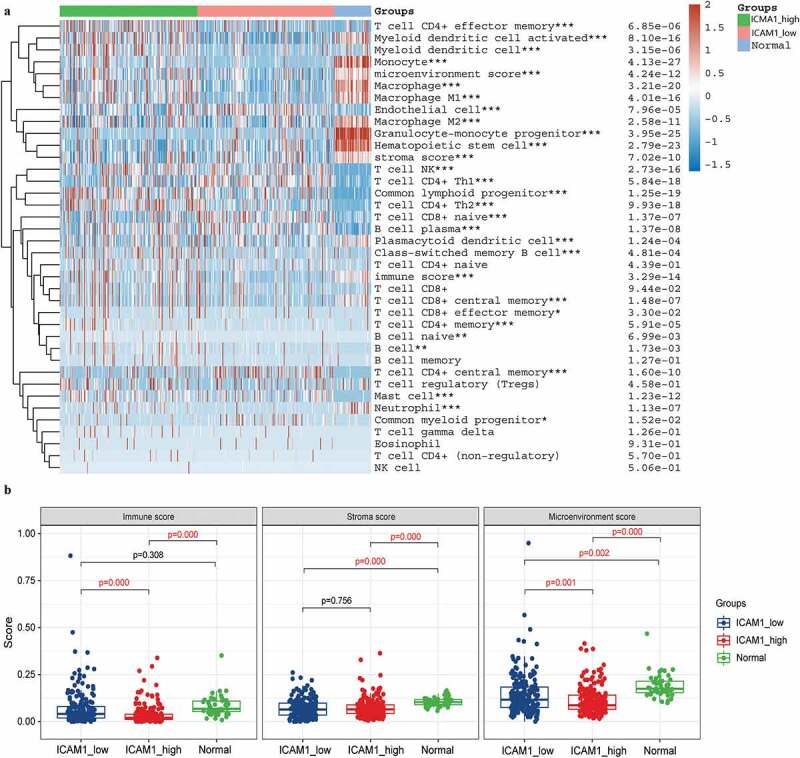
Immune cell infiltration analysis in ICAM-1_high, ICAM-1_low and normal samples. (a) The percentage abundance of tumor-infiltrating immune cells was analyzed by the xCell algorithm using the immunodeconv package and visualized by the ggplot2 and pheatmap packages in R. The significance of the three groups passed the Kruskal-Wallis test. (b) The immune score, stromal score and microenvironment score were visualized by the ggplot2 package. The significance of the three groups passed one-way ANOVA. P < 0.05 was defined as statistically significant

Previous studies have suggested that AFP and ICAM-1 might become potential immunotherapy targets. To evaluate this, we first divided the 371 HCC samples into a high expression group and a low expression group according to the median values of AFP and ICAM-1 expression. Then, the xCell algorithm was employed to assess the percentage abundance of 35 tumor infiltrating immune cells among the normal group, high expression group and low expression group. We found that 28 of 35 immune cells were significantly different among the AFP_high, AFP_low and normal groups (P < 0.05, Figure 5(a)), and the immune score and stromal score were significantly different between the AFP_high group and AFP_low group (P < 0.05, Figure 5(b)). Moreover, similar results with the percentage of immune cells were observed in the ICAM-1_high, sICAM-1_low and normal groups (Figure 6(a)), and the immune score and microenvironment score were significantly different between the ICAM-1_high group and the ICAM-1_low group (P < 0.05, Figure 6(b)). The immune score and stroma score have been proven to be of great value for tumor diagnosis and prognostic evaluation, and patients with favorable prognosis tend to have high stromal scores, high immune scores, and high immune infiltration [22]. Collectively, the AFP_low group and ICAM-1_low group possessed higher immune scores and stromal scores, suggesting that the patients in these groups might be associated with a favorable prognosis, which was consistent with our clinical results.

**Figure 7.**
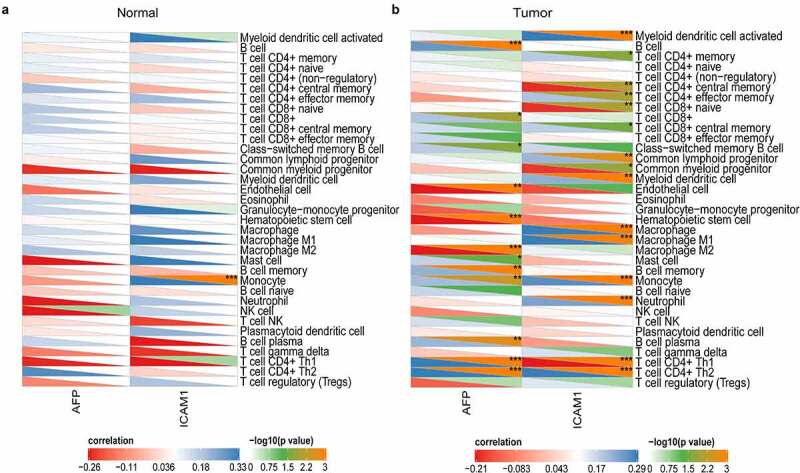
Correlation analysis of AFP and ICAM-1 with multiple immune cells. The correlation heatmap of AFP and ICAM-1 with immune cells was plotted by the psych package in the normal samples (a) and HCC samples (b), and the correlation was calculated by Pearson’s correlation. P < 0.05 was defined as statistically significant

In addition, the correlation of the expression of AFP and ICAM-1 to immune cells was analyzed by Pearson correlation in the normal and tumor samples. In normal samples, AFP was not significantly related to all 35 immune cells, and ICAM-1 was also only significantly associated with monocytes (Figure 7(a)). However, in the tumor samples, AFP (12/35) and ICAM-1 (15/35) were significantly associated with numerous immune cells, such as macrophages, monocytes, and T cells (Figure 7(b)). This suggested that AFP and ICAM-1 were correlated with multiple immune cells in tumor samples but not in normal samples, which contributed to the immunotherapy of HCC.

### Prediction of AFP and ICAM-1 in immunotherapy response

**Figure 8.**
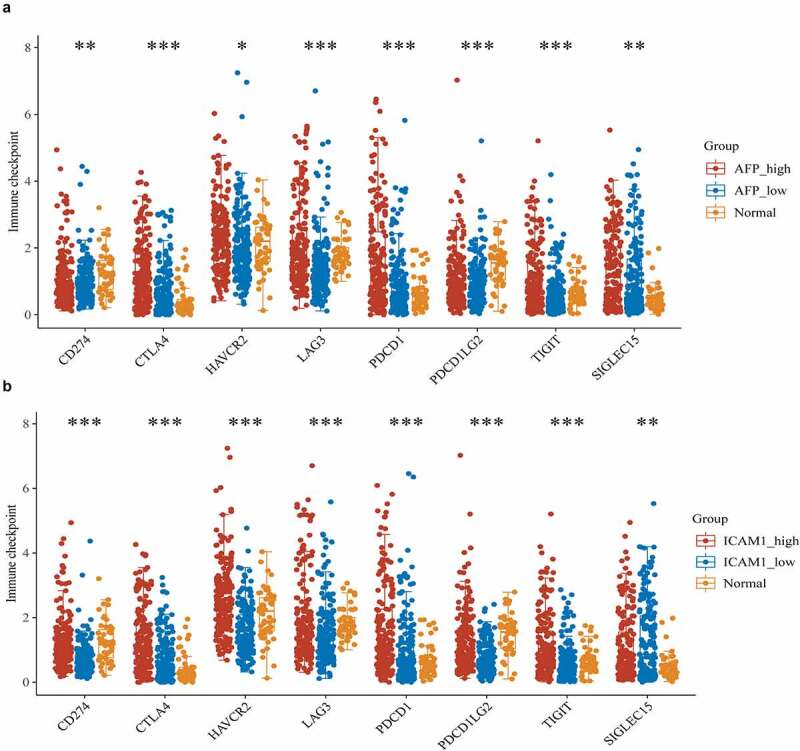
Immune checkpoint analysis. The expression of immune checkpoint-relevant genes in AFP_high, AFP_low and normal samples (a) or ICAM-1_high, ICAM-1_low and normal samples (b), including SIGLEC15, IDO1, CD274, HAVCR2, PDCD1, CTLA4, LAG3 and PDCD1LG2, was analyzed and visualized by the ggplot2 package in R. The significance of the three groups passed the Kruskal-Wallis test. P < 0.05 was defined as statistically significant

Immune checkpoint molecules are expressed on immune cells and will inhibit the function of immune cells so that the body cannot produce an effective antitumour immune response, and the tumor forms an immune escape [23]. Emerging ICB therapy precisely suppresses the function of immune checkpoint molecules to ease or even treat cancer, which has shown astounding outcomes with the potential to improve disease conditions in advanced cancers [24]. To evaluate the correlation of AFP and ICAM-1 to immune checkpoint molecules, we selected SIGLEC15, TIGIT, CD274, HAVCR2, PDCD1, CTLA4, LAG3, and PDCD1LG2 as immune checkpoint-relevant transcripts to analyze the expression differences of these molecules among the high expression, low expression and normal groups. The expression of these molecules was significantly different in the AFP_high, AFP_low and normal groups (P < 0.05, Figure 8(a)) and in the ICAM-1_high, ICAM-1_low and normal groups (P < 0.05, Figure 8(b)), suggesting that APF or ICAM-1 was closely related to these immune checkpoint molecules.

**Figure 9.**
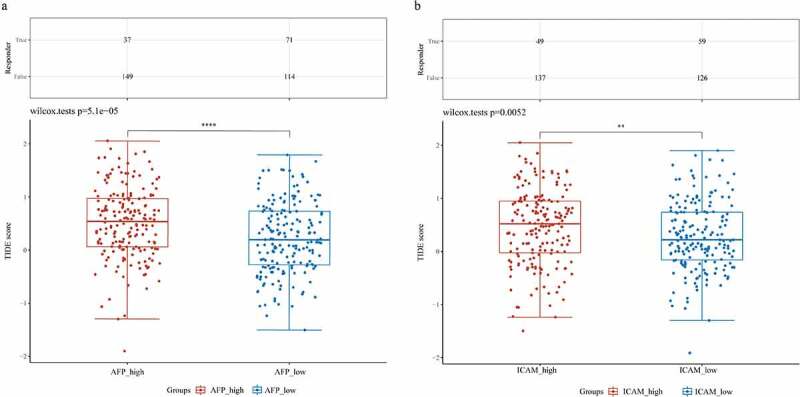
Prediction of immunotherapy response. ICB response prediction and TIDE scores were analyzed by the ImmuCellAI and TIDE algorithms in AFP_high and AFP_low samples (a) or ICAM-1_high and ICAM-1_low samples (b). The significance of the two groups passed the Wilcox test. P < 0.05 was defined as statistically significant

The subsequent analysis involved ICB therapy, and we employed the TIDE algorithm to predict the responsiveness of the samples in the high expression group and low expression group to immune checkpoint inhibitors. We found that the patients in the AFP_high group possessed higher TIDE scores than those in the AFP_low group (Wilcox test P = 5.1e-05, Figure 9(a)). Similarly, the patients in the ICAM-1_high group had higher TIDE scores than those in the ICAM-1_low group (Wilcox test P = 0.0052, Figure 9(b)). These results suggested that the patients in the AFP_high group and ICAM-1_high group had poor efficacy of ICB therapy and had a short survival period after receiving ICB treatment, which was consistent with our clinical results and the immune score results.

## Discussion

The aim of this study was to explore the potential value of AFP and ICAM-1 on the prognostic prediction and immunotherapy of HCC. AFP and sICAM-1 are specific indicators used to diagnose liver cancer or evaluate tumor invasiveness. AFP has been of great clinical value in both preoperative and postoperative surveillance for liver cancer, and has been used to clinically and serologically diagnose HCC for more than 20 years [25]. The ICAM-1 is a glycoprotein that induces intercellular connections and interactions, which physiologically are mainly expressed on the surface of various cells, such as macrophages and endothelial cells, but not in normal liver tissues [12]. The sICAM-1 is different from ICAM-1. ICAM-1 is divided into sICAM-1 (soluble type) and mICAM-1 (membrane type). The sICAM-1 represents a circulating form of ICAM-1 that is constitutively expressed or is inducible on the cell surface of different cell lines [26]. Detection of serum sICAM-1 level can reflect the local expression of ICAM-1. Previous studies had confirmed the clinical diagnosis value of AFP and sICAM-1 in HCC, but the combined effect of AFP and sICAM-1 has not been evaluated, and whether they had other potential value still needs to be further explored.

Our analysis on the long-term recurrence rates and OS rates of patients with different AFP and sICAM-1 levels showed that the patients with AFP > 200 μg/L or sICAM-1 > 2000 μg/L had lower TFS rates and OS rates than the patients with AFP < 200 μg/L or sICAM-1 < 2000 μg/L in primary HCC. However, the study of Shimura *et al* showed HCC patients with sICAM-1 ≥ 440 ng/ml exhibited a poorer OS and recurrence-free survival than those with sICAM-1 < 440 ng/ml (P = 0.002) [27]. Besides, a study in gastric cancer showed that the median OS and progression-free survival in patients whose AFP level decreased by ≥ 50% were respectively 32.0 (4–74) and 24.0 (1–66) months, and which in patients whose AFP level decreased by < 50% were respectively 12.5 (0–69) and 9.0 (0–63) months [28]. These results suggested that, although the cutoff values of AFP and sICAM-1 levels were inconsistent which might be due to the small sample volume, the higher AFP and sICAM-1 levels were closely associated with a poorer prognosis of patients. Therefore, the AFP and sICAM-1 levels in prognosis evaluation of HCC still need to be further explored with expanded sample volume before they could be used in clinical practice.

After surgical treatment, variations in the AFP and sICAM-1 levels of patients were not completely unanimous. The patients in the Group UI had the worst prognosis, followed by Group D, and the patients Group N had the best prognosis. These results suggested that, regardless of whether the preoperative AFP and sICAM-1 levels were normal or higher than normal, as long as the postoperative AFP and sICAM-1 levels return to normal and remain stable, the patients would have favorable prognosis. Moreover, when preoperative AFP > 20 μg/L and sICAM-1 > 1000 μg/L, the patient had a poorer prognosis, suggesting that the combined application of these two indicators before surgery could predict patient prognosis. At present, there were almost no studies on exploring in depth the effects of preoperative and postoperative AFP and sICAM-1 levels on the prognosis evaluation of HCC patients. Therefore, in our study, although the value of preoperative AFP and sICAM-1 levels still needs to be explored with extensive sample volume in the prognosis prediction, the prognosis of the patient could be predicted based on the relative change of preoperative and postoperative AFP and sICAM-1 levels of patient. We hoped that our study could provide some guidance for the clinic.

Additionally, we employed the RNA sequencing data and clinical information of TCGA-LIHC datasets to validate the accuracy of AFP, ICAM-1 and their combination in the diagnosis and prognostic prediction of HCC. We found that the combined application of AFP and ICAM-1 showed higher accuracy than their single application in both the diagnosis (AUC: 0.783_combination_ vs. 0.720_AFP_ vs. 0.504_ICAM-1_) and prognostic prediction (AUC: 0.659_combination_ vs. 0.507_AFP_ vs. 0.557_ICAM-1_) of HCC, which was in line with our abovementioned clinical research results. A study suggested that combining the detection of AFP with new diagnostic markers increased both the sensitivity and specificity to diagnose HCC [29]. For example, AFP combined with either the lens culinaris lectin or the Golgi protein 73 could increase the diagnostic specificity, sensitivity and accuracy rate, greatly improving the early diagnosis rate of liver cancer [30,31], which was consistent with our findings. New combined biomarkers needed to be continuously discovered to improve the diagnostic effect of AFP, and the combination of AFP with ICAM-1 possesses high accuracy in both the early diagnosis and prognostic prediction of HCC and was expected to provide new credible indicators for the diagnosis of HCC.

In addition to being an oncofoetal antigen and diagnostic indicator for liver cancer, AFP also had multifarious biological functions, such as regulation of cell differentiation, proliferation, and tumorigenesis [11], and in fact, AFP could be used as an immunotherapeutic target for HCC [13]. To evaluate this, we first analyzed the relationship of AFP or ICAM-1 and the TME by bioinformatics methods and found that both AFP and ICAM-1 had a significant correlation with multiple immune cells, including CD4 + T cells, macrophages, and monocytes, in HCC samples but not in normal samples. In addition, the samples with low AFP expression or low ICAM-1 expression had higher immune scores and stromal scores, which were used to evaluate immune cells and stromal cells in the TME. Immune cells and stromal cells are the two main types of nontumour components, and these nontumour cells dilute the purity of tumor cells and have been proven to be of great value for tumor diagnosis and prognostic evaluation [32]. High immune scores and stromal scores are associated with a favorable cancer prognosis, including HCC [33]. Therefore, the patients with low expression of AFP or ICAM-1 were likely to have a favorable prognosis, while the patients with high expression of AFP or ICAM-1 were likely to have a poorer prognosis in our study, which was consistent with our clinical findings.

Due to AFP and ICAM-1 were connected to a variety of immune cells, thus we speculated whether they could influence immunotherapy for HCC. We analyzed the expression of immune checkpoint molecules and found that the expression of TIGIT, HAVCR2, PDCD1, CTLA4, and LAG3 in the AFP_high group was significantly higher than that in the AFP_low group, while only the expression of HAVCR2 in the ICAM-1_high group was significantly higher than that in the ICAM-1_low group. The expression of other checkpoint molecules was not significantly different between the groups. Perhaps, HAVCR2 might be a target in ICB therapy, making patients with low expression of AFP combined with ICAM-1 obtain a favorable treatment effect. Some studies had proved our speculation, the TIM-3 protein encoded by the HAVCR2 gene should be used as a target for immunotherapy of HCC [34,35]. These results suggested that AFP and ICAM-1 might be used to distinguish the patients who responded to ICB therapy (such as HAVCR2 blockade), but this still needs further clinical research.

Subsequent ICB response prediction analyses also confirmed that the patients in the AFP_low group and ICAM-1_low group had a lower TIDE score, suggesting that these patients had a favorable efficacy for ICB therapy, and their survival period was long after receiving ICB therapy, which was consistent with above results.

## Conclusions

Our study confirmed that the patients whose AFP and sICAM-1 levels were normal before surgery or recovered to normal after surgery had preferable OS. Moreover, AFP and ICAM-1 combination application showed higher accuracy in both the diagnosis and prognostic prediction than alone in HCC, and the patients with low-expression of the two indicators were most likely to benefit from ICB therapy. These results suggest that AFP and ICAM-1 were considered as prognostic predictors, and might have a vital role in immunotherapy of HCC.

## Abbreviation

hepatocellular carcinoma (HCC); tumor free survival rate (TFS); overall survival rate (OS); α-fetoprotein (AFP); soluble intercellular adhesion molecule-1 (sICAM-1); the cancer genome atlas-liver hepatocellular carcinoma (TCGA-LIHC); area under the curve (AUC); receiver operating characteristic curve (ROC); tumor size/lymph nodes/distance metastasis (TNM); immune checkpoint blockade (ICB); least absolute shrinkage and selection operator (LASSO); tumor immune dysfunction and exclusion (TIDE); statistic package for social science (SPSS); analysis of variance (ANOVA); Student-Newman-Keuls(SNK); tumor microenvironment (TME).
